# Ultrasound-enhanced electrospinning

**DOI:** 10.1038/s41598-018-22124-z

**Published:** 2018-03-13

**Authors:** Heikki J. Nieminen, Ivo Laidmäe, Ari Salmi, Timo Rauhala, Tor Paulin, Jyrki Heinämäki, Edward Hæggström

**Affiliations:** 1 Electronics Research Laboratory, Department of Physics, University of Helsinki, Helsinki, Finland; 2 Medical Ultrasonics Laboratory (MEDUSA), Department of Neuroscience and Biomedical Engineering, Aalto University, Espoo, Finland; 3 Institute of Pharmacy, University of Tartu, Tartu, Estonia; 4 Department of Immunology, Institute of Biomedicine and Translational Medicine, University of Tartu, Tartu, Estonia

## Abstract

Electrospinning is commonly used to produce polymeric nanofibers. Potential applications for such fibers include novel drug delivery systems, tissue engineering scaffolds, and filters. Electrospinning, however, has shortcomings such as needle clogging and limited ability to control the fiber-properties in a non-chemical manner. This study reports on an orifice-less technique that employs high-intensity focused ultrasound, *i.e*. ultrasound-enhanced electrospinning. Ultrasound bursts were used to generate a liquid protrusion with a Taylor cone from the surface of a polymer solution of polyethylene oxide. When the polymer was charged with a high negative voltage, nanofibers jetted off from the tip of the protrusion landed on an electrically grounded target held at a constant distance from the tip. Controlling the ultrasound characteristics permitted physical modification of the nanofiber topography at will without using supplemental chemical intervention. Possible applications of tailor-made fibers generated by ultrasound-enhanced electrospinning include pharmaceutical controlled-release applications and biomedical scaffolds with spatial gradients in fiber thickness and mechanical properties.

## Introduction

Electrospinning (ES) is nanotechnology that employs an electric field to pull nanofibers from a polymer solution. A Taylor cone^1,2^ is formed at the surface of the polymer solution extrusion, typically charged with high voltage (several -kV), and it serves as the location of the ejection of an initial fiber. The final fiber is typically formed through a drying process. The product is collected onto a charged collector by electric attraction (typically higher potential than at the polymer, *e.g*. ground or positive potential). Different implementations of ES exist featuring different potentials at the source polymer and target^3^.

In conventional ES, fiber ejection typically takes place from a small polymer droplet located at an orifice, *e.g*. at a tip of a charged needle. The droplet is refilled by pumping new polymer solution to the needle tip through the needle. This is widely used approach, but is limited by needle clogging issues^4^, and rapid control of fiber properties during an ES event has not been reported. Clogging can be overcome with orifice-less approaches^5–12^. However, changing the fiber-properties (*e.g*. the diameter) rapidly during ES is limited to changing common ES parameters^13^, the distance between the Taylor cone and the collector^14^, the environment^15,16^, nozzle diameter^17^, polymer solution properties^18,19^, and solution feed-rate^20^. All these approaches are impractical and limit the spatiotemporal control. Sub-second modulation of fiber the diameter could permit generating layer-by-layer fibers with different thickness/shape properties. This could potentially enable gradients in fiber constructs, for purposes such as (i) controlled drug release systems, (ii) tissue engineering scaffolds, and (iii) filters.

High-intensity focused ultrasound (HIFU) provides a means to actuate matter. In practice, ultrasound (US) with high intensity can be used to push or palpate material and material interfaces using acoustic radiation pressure^21^. At interfaces between gas, liquid, and solid, US can generate radiation forces that push the boundary. In a configuration, where US travels through liquid to a liquid-gas interface (*e.g*. polymer solution-air -interface), a localized impingement of US on this acoustic interface creates a protrusion at the liquid surface. Inside fluids, a travelling US wave can generate acoustic streaming along the direction of sound propagation^22^. In conjunction with bubbles, US can make micro/nano-bubbles oscillate and, contribute to rapid streaming^23,24^ and fluid micro-jets at micron scale^25,26^. HIFU can also heat a material within a confined volume, *e.g*. within “ultrasonic fountains” generated at liquid interface^27^, induced by acoustic radiation force. While US-matter interactions are many, their exploitation to manipulate the electrospun fiber during ES has been overlooked in the literature.

To date, US in conjunction with ES, has not been systematically investigated as a means to modify the topography or diameter of nanospun fibers. This study aims to *(i)* to establish an orifice-less ES system based on acoustic radiation pressure, and *(ii)* to explore the technique’s potential to modify the topography of polyethylene oxide (PEO) nanofibers.

## Results

**Figure 1:**
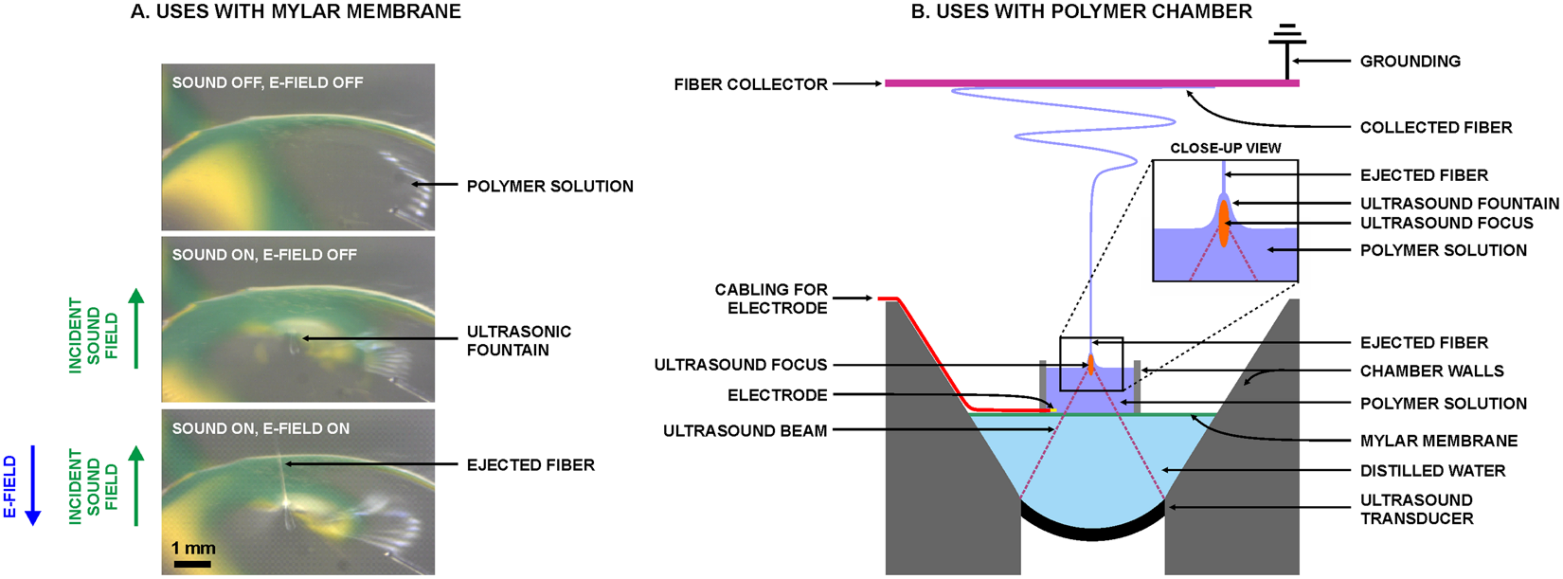
(**A**) The applied high-intensity ultrasound generated an acoustic fountain, when a polymer droplet was placed on a 0.2-mm thick mylar membrane (acoustically conducting, but electrically isolating), while focusing the beam through water at the polymer-air interface. When the polymer was charged using an electrode (from −5 kV to −15 kV) and an electrically grounded target was placed above the fountain, a liquid jet was observed at the top of the ultrasonic fountain. (**B**) The method described in (**A**) was developed into a USES system, which was employed for systematic investigation of ultrasonic fiber-topography control using an −11.3 kV electrode voltage and 15 cm polymer to target distance.

The ultrasound-enhanced electrospinning (USES) method (Fig. 1A) and a system (Fig. 1B) were successfully established. High-intensity US was generated by a focused piezo-ceramic transducer. The US beam (focus width = 1.0 mm, focus length = 6.9 mm at −3dB of peak-positive-pressure) was directed through a 0.2-mm thick mylar membrane (beam axis perpendicular to the mylar membrane; the membrane is acoustically conducting, but electrically isolating) to a location within an open chamber carrying the source polymer solution (Fig. 1B). The space between the membrane and transducer was filled with purified water to conduct sound from the transducer to the target polymer. When the polymer-air -interface level was vertically adjusted to coincide with the US focus, a visible protrusion of polymer (“ultrasonic fountain”) with a height up to few millimeters was observed for specific ultrasonic parameters (time-averaged total acoustic power, *i.e*. TAP = low: 0.3 W; medium: 1.0 W; high: 1.4 W; frequency = 2.16 MHz; pulse repetition frequency, *i.e*. PRF = 150; duty cycle = 1.7%). When an electrode inside the polymer chamber, but outside the US beam, was charged (−11.3 kV), vertical ejection of polymer was visually observed from the tip of the protrusion. The ejected fibers were collected on an aluminium foil target, which was electrically grounded and positioned horizontally 15 cm above the polymer solution surface.

**Figure 2:**
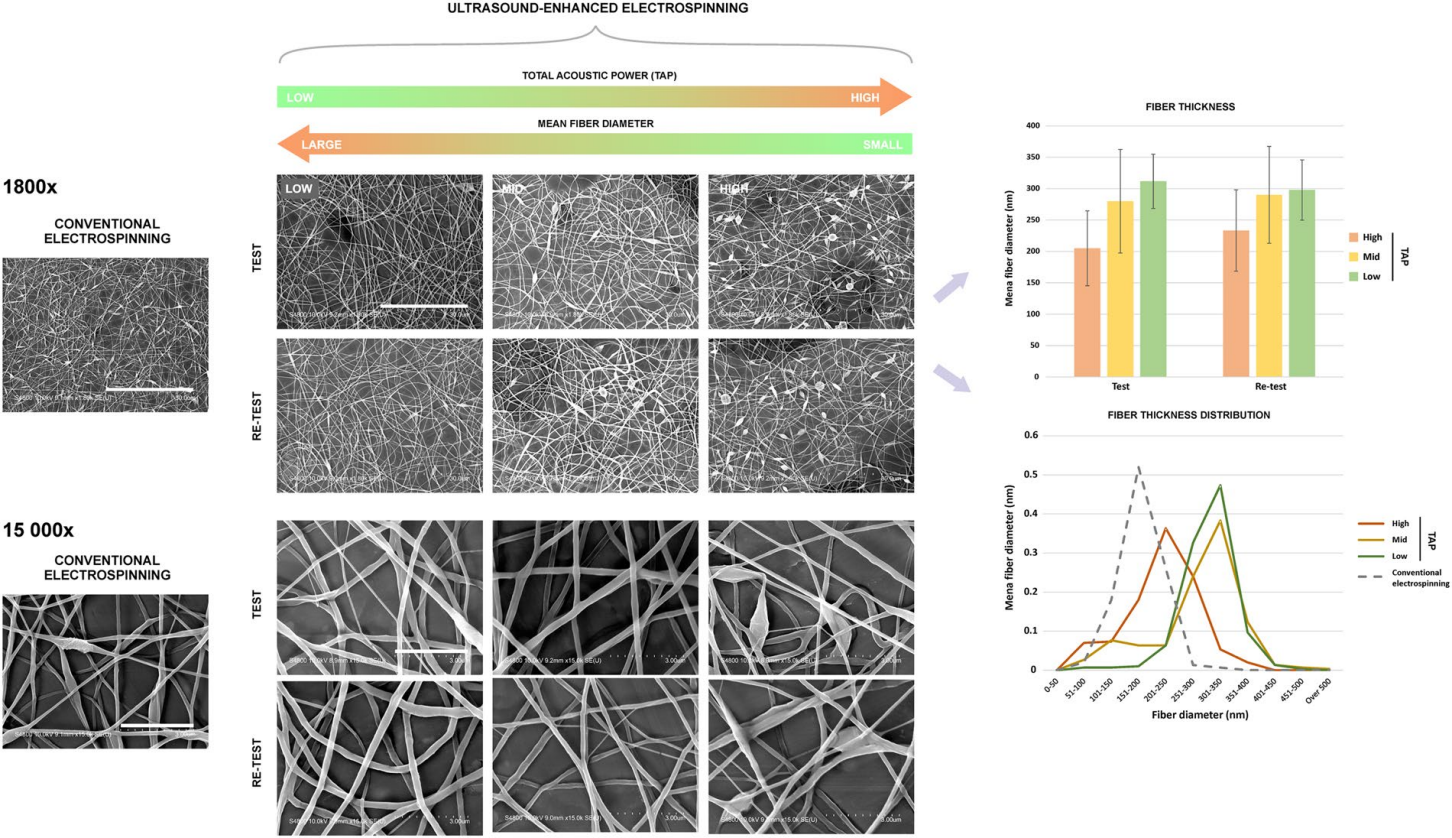
Left and center: Scanning electron microscope images of fibers produced with the ultrasound-enhanced electrospinning (USES) device at low, mid and high ultrasonic power. Top row corresponds to the test and the lower row to a re-test. Low ultrasonic power exhibited fibers with minor “beading”, whereas beading was most pronounced in fibers generated with high ultrasound power and with the reference technique (conventional electrospinning). The images suggest that the visual appearance of fibers was similar in the test compared to the re-test. Ultrasonically enhanced fibers appear thicker than those produced with the reference method. The results suggest that the appearance of the topography of fibers generated with USES can be repeatedly modified. Right: Distribution of fiber diameters produced at high, mid or low ultrasound power in test/re-test -experiments. All methods produced nanofibers with 50–500 nm diameter. Ultrasound-enhanced fibers were qualitatively thicker and statistically different in diameter (*p* < 0.0001) than those produced with the reference method. The fibers produced using high ultrasound power were qualitatively thinner and statistically significantly different (*p* < 0.0001) than those produced with ‘mid’ or ‘low’ ultrasound power. The ‘mid’ ultrasound exposure produced a narrower fiber distribution than the low power, but low power produce thinner fibers in the 50–200 nm range. The results suggest that the distribution in fiber diameter can be modified by changing the ultrasound power, without changing the chemical composition of the source polymer. The reference fibers were produced with conventional needle electrospinning. For comparability the area of each histogram was normalized to 1. For statistical analysis, non-parametric Kruskall-Wallis for pairwise comparison with Bonferroni adjustment was used. Scale bars for 1800× and 15 000× magnifications corresponds to 30 μm and 3 μm, respectively.

USES provided means to produce stable nano-fiber production. Repeatedly, the produced nanofiber constructs were visually the same, when same TAP was applied. Low (TAP = 0.3 W) and medium (TAP = 1.0 W) US power produced on average thicker fibers than those produced with the high US power setting (Fig. 2). The ‘medium’ and ‘high’ power as well as the reference method (conventional ES) produced more beads (ellipsoidal or round thickenings within the fiber constructs) than the ‘low’ power setting (Fig. 2).

Quantitative analysis of the fibers in a test/re-test -experiment demonstrated that on average the thinnest fibers were produced with the reference method (178 ± 64 nm; mean ± S.D; *n* = 150) and repeated with USES using the ‘high’ US power setting (test: 205 ± 60 nm, *n* = 150; re-test: 233 ± 65 nm, *n* = 150; mean of the two tests = 219 nm) (Fig. 2, right). The average fiber diameters for both medium and low US power settings were on average higher (medium; test: 280 ± 82 nm, *n* = 150; re-test: 290 ± 77 nm; mean of the two tests = 285 nm; Low; test: 312 ± 43 nm, *n* = 150; re-test: 298 ± 48 nm, *n* = 150; mean of the two tests = 305 nm) (Fig. 2, right). The fiber diameters in each setting group differed statistically significantly: low-mid, *p* = 0.0182; low-high, *p* < 0.0001, mid-high, *p* < 0.0001. The fiber diameters in each setting group also differed from fiber diameters in reference group (ES-generated fibers) (*p* < 0.0001).

## Discussion

The new orifice-less US-enhanced ES method, USES, was successfully established in this study. USES demonstrated capability to generate polymeric nanofibers, whose properties were associated with the applied US settings in a repeatable manner. The data in this manuscript was generated from spinning events that lasted 38 to 60 seconds. However, the method permitted successful spinning for hours, when a capillary was connected to the source polymer chamber to continuously re-fill the polymer chamber with PEO. In this arrangement, the feed-rate was up to 3 mL/h, which is 3× compared to the feed-rate 1 mL/h of the conventional ES.

Interestingly, the mean fiber diameter decreases with increasing TAP (Fig. 2, right). The distribution of fiber diameters is also affected by the applied ultrasonic settings (Fig. 2, right) and the groups differed statistically. It appears that the fibers generated with USES are thicker than those generated with conventional ES using the same polymer solution.

Several phenomena may take place at the proximity of the ultrasonic fountain, and may be associated with the demonstrated control of fiber properties:

1. Acoustic radiation force^21^ in conjunction with the electric field^1,2^
2. Capillary waves at the surface of the polymer generated by US^28,29^
3. Cavitation^23–25,28^,
4. Acoustic streaming^22^
5. Thermal effects^27^.

Since the *(i)* ultrasonic fountain generation is reported in the literature^28,30^, and since *(ii)* fiber generation was impossible without the ultrasonic fountain, and since *(iii)* fiber ejection was observed at the tip of the fountain, the acoustic radiation force plays a fundamental role in determining where the fiber generation occurs. The electric field density typically concentrates at material extrusions; therefore, it is expected to concentrate also at the tip of the fountain. In our experience, if US settings with visible acoustic streaming were used, the sharpness of the protrusion was compromised, which prevented spinning from starting. Spinning occurred, when the protrusion was narrow, *i.e*. ~1 mm in diameter or less.

Interestingly, the fibers generated with ES using the same polymer were on average smaller in diameter compared to those generated with USES. One potential explanation for this is the self-focusing funnel-like shape of the ultrasonic fountain. This is expected to guide US waves towards the fiber and potentially into the fiber. This focusing may, therefore, induce upwards momentum also in the fiber in addition to that momentum generated by the electric field. This addition would enhance mass transfer, as suggested by the higher feed-rate (up to 3 mL/h) compared to ES (1 mL/h), and, thus, produces thicker fibers. Moreover, temperatures at ultrasonic fountains can be high due to high ultrasonic intensities^27^; therefore, thermal effects are expected to contribute to *e.g*. the drying process of the fiber and possibly to modification of the polymer structure. Ultrasound has also been reported to be able to modify the polymer viscosity or conductivity^11,31^. Further investigations specifically for each polymer-drug -combination, would be deemed, because of potential damage to the molecule or polymer that could affect the drug activity.

In our experiments, the initiation of ES from the ultrasonic fountain strongly depended on the pulse repetition frequency (PRF). Typically, ES initiation occurred for 100–200 Hz PRF; the empirical optimum PRF was 150 Hz for PEO (3% w/v). Moreover, for ES using lowest TAP setting (generates a smaller fountain than with the mid or high TAP setting), higher TAP was initially required to initiate spinning. Once spinning began, the US settings could be changed, while maintaining fiber production even at low TAP.

The possible applications of USES-generated fibers are many. In the following we discuss two applications that we find prominent.

1. Electrospun fibers are investigated as possible means to modify drug release, especially poorly water-soluble ones. The fiber diameter is associated with the total active surface area per unit mass. Thin fibers release drugs faster than thick fibers^32^. Therefore, the capability to modify fiber diameter by physical means provides potentially a way to tailor drug release properties within the same fiber construct non-chemically, even though it was not demonstrated in this study. This would be a great advantage compared to the state-of-the-art, which typically requires the chemical modification of the polymer solution (*i.e*. changing concentration or nature of the polymer) of the polymer solution to modify fiber properties such as topography and diameter.
2. Electrospun fibers are widely used to produce scaffolds for tissue engineering purposes^33^. Tissue engineering using *e.g*. stem cells differentiate by reacting to their mechanical environment^33,34^). A scaffold with gradients in fiber construct properties should influence the mechanical environment the cells are embedded in^33,35^; therefore, USES-induced gradients in fiber construct properties could provide unique means to produce tissue engineering scaffolds with gradients in mechanical properties simply by changing the US parameters during the spinning process.

The literature reports on several orifice-less ES techniques^5–10^. Unfortunately, none of the available approaches have been reported to be capable of controlling fiber topography or diameter spatiotemporally in a time-frame of a second. Therefore, USES is a significant improvement over the pre-existing methods. Moreover, USES can be scaled up: (i) one can multiply the number of transducers and ES jets; or (ii) a transducer array can be used to electronically steer the focus to several positions on a polymer surface to induce several ultrasonic fountains with several locations of fiber ejection within a single source polymer chamber.

To conclude, we established a new orifice-less ES platform that features US enhancement. USES provides physical means to control polymeric fiber topography in PEO and diameter without supplementary chemical intervention. It also solves clogging issues associated with conventional ES approaches. USES could be scaled up if found feasible for industrial use. Possible applications include, but are not limited to, tailored drug delivery systems and tissue engineering scaffolds with gradients.

## Materials and Methods

### Experimental system

An ES setup illustrated in Fig. 1B was established. The US transducers were electrically grounded from its top surface and charged from its bottom surface with a driving voltage. The signal driving the piezoceramic transducer (radius of curvature = 29 mm, outer diameter = 29 mm) was provided by a signal generator and a power amplifier (500A100A, Amplifier Research Corp., Souderton, PA). A high voltage supply (P3067D; Kevex Instruments, San Carlos, CA) was used to charge the electrode within the polymer chamber (Fig. 1B).

### Electrospinning

The solution for ES was PEO aqueous solution (3% w/v), prepared by overnight dissolving PEO (Average Mv 900,000; Sigma-Aldrich Inc., St. Louis, MO) into deionized water with agitation in ambient conditions. An in-house conventional ES system with a syringe, pump (KDS-230-CE; KD Scientific Inc., Holliston, MA) and a 23 G hypodermic needle, which served as the polymer feeding capillary (feeding rate was 1 ml/h), was used to produce reference nanofibers, otherwise with same settings as in USES.

Three different ultrasonic settings were used in the USES (see Results). Tests with different US settings were repeated twice with same settings, but with reversed order. Control fibers were generated with conventional ES. Fiber collection time was 38 sec to 1 min. Experiments were done in ambient conditions. To avoid influence of *e.g*. change in relative humidity all spinning experiments were done inside one hour. The samples for ES and USES were generated from the same starting aqueous PEO solution.

### Scanning electron microscopy (SEM) and fiber analysis

From each test, two samples were selected for SEM analysis (S-4800 FE-SEM; Hitachi Ltd., Tokyo, Japan). The samples were attached to the sample holder with carbon tape (Carbon Conductive Double-faced Adhesive Tape; Nisshin EM Co. Ltd., Tokyo, Japan) followed by platinum-coating (6 nm) prior to SEM imaging with 1 800× and 15 000× magnifications (acceleration voltage = 10 kV, emission current = 10 µA). The diameters of nanofibers were measured using ImageJ 1.51 g software at random locations within the SEM images. The fiber diameter is presented as mean (±SD) of 150 individual measurements from each spinning event.

### Statistical analysis

For comparison of fiber diameter distributions in the experimental groups, a non-parametric Kruskall-Wallis for pairwise comparison with Bonferroni adjustment was used. Statistical analysis was performed using the computing environment R (R version 3.4.0, R Development Core Team, 2017).
